# Screening of *Bauhinia purpurea* Linn. for analgesic and anti-inflammatory activities

**DOI:** 10.4103/0253-7613.51345

**Published:** 2009-04

**Authors:** C.S. Shreedhara, V.P. Vaidya, H.M. Vagdevi, K.P. Latha, K.S. Muralikrishna, A.M. Krupanidhi

**Affiliations:** Department of Pharmacognosy, Manipal College of Pharmaceutical Sciences, Manipal - 576 104, India; 1 Department of Chemistry, Jnana Sahyadri, Shankaraghatta - 577 451, India; 2 Bapuji Pharmacy College, Davanagere - 577 004, India

**Keywords:** Analgesic activity, anti-inflammatory activity, *Bauhinia purpurea* Linn, eddy's hot plate

## Abstract

**Objectives:**

Ethanol extract of the stem of *Bauhinia purpurea* Linn. was subjected to analgesic and anti-inflammatory activities in animal models.

**Materials and Methods:**

Albino Wistar rats and mice were the experimental animals respectively. Different CNS depressant paradigms like analgesic activity (determined by Eddy's hot plate method and acetic acid writhing method) and anti-inflammatory activity determined by carrageenan induced paw edema using plethysmometer in albino rats) were carried out, following the intra-peritoneal administration of ethanol extract of *Bauhinia purpurea* Linn. (BP) at the dose level of 50 mg/kg and 100 mg/kg.

**Results:**

The analgesic and anti-inflammatory activities of ethanol extracts of BP were significant (*P* < 0.001). The maximum analgesic effect was observed at 120 min at the dose of 100 mg/kg (i.p.) and was comparable to that of standard analgin (150 mg/kg) and the percentage of edema inhibition effect was 46.4% and 77% for 50 mg/kg and 100 mg/kg (i.p) respectively. Anti-inflammatory activity was compared with standard Diclofenac sodium (5 mg/kg).

**Conclusion:**

Ethanol extract of *Bauhinia purpurea* has shown significant analgesic and anti-inflammatory activities at the dose of 100 mg/kg and was comparable with corresponding standard drugs. The activity was attributed to the presence of phytoconstituents in the tested extract.

## Introduction

*Bauhinia purpurea* (Leguminosae) is a medium sized deciduous tree, sparingly grown in India. This plant is used traditionally in dropsy, pain, rheumatism, convulsions, delirium, and septicemia.[1] The bark of the plant is used as an astringent in the treatment of diarrhea. Its decoctions are recommended for ulcers as a useful wash solution.[2] The aerial parts of the plant are reported to contain flavone gylcosides, foliar flavonoids, 6-butyl-3-hydroxy flavanone, amino acids, phenyl fatty ester, lutine and β-sitosterol.[3–8] These active constituents have been attributed the therapeutic activity of the plant. Therefore, the present study was undertaken to evaluate their analgesic and anti-inflammatory activities.

## Materials and Methods

### Preparation of the extract

Coarse powder of the air-dried bark was subjected to successive solvent extraction method using solvents of increasing polarity [petroleum ether – (60-80°C), chloroform, ethanol at its boiling temperature] in a Soxhlet extraction unit till exhaustion and finally aqueous extract was prepared using chloroform water by simple maceration at room temperature. Each aqueous extract was carefully evaporated in a rotary evaporator under controlled temperature and reduced pressure to get the extract and the yield and percentage yield of various extracts is shown in Table 1.

**Table 1:** Yield, color and consistency of various extracts of *Bauhinia purpurea* Linn. (BP)

*Extract*	*Yield (%) w/w*	*Color*	*Consistency*
Petroleum ether	0.9	Dark brown	Semisolid
Chloroform	0.6	Dark brown	Amorphous powder
Ethanol	4.5	Reddish brown	Powder
Aqueous	6.6	Reddish brown	Powder

### Phytochemical screening[9–11]

Each extract was subjected to phytochemical screening and the preliminary chemical examination of ethanol extract revealed the presence of steroids, flavonoids, tannins, coumarins, carbohydrates and reducing sugars. Flavonoids exhibit varied biological activities that include analgesic, anti-inflammatory, antioxidant, hepatoprotective and anti-ulcer activities. Tannins are protectants. Based on this, it was contemplated to carry out the screening of ethanolic extract for analgesic, anti-inflammatory activities. Petroleum ether extract and chloroform extract revealed the presence of steroids and hence these were not tested. The results are compiled in Table 2.

**Table 2:** Phytochemical investigation[9–11] of various extracts of *Bauhinia purpurea*

*Extracts*	*Constituents investigated*
Petroleum ether	Steroids
Chloroform	Steroids, free anthraquinones
Ethanol	Steroids, flavonoids, tannins, coumarins, carbohydrates and reducing sugars
Aqueous	Flavonoids, tannins, carbohydrates and reducing sugars

### Animals

Albino Wistar rats and mice of either sex weighing 150-200 gm and 25-30 gm were bred and maintained under standard conditions in the central animal house at the college and animal ethical committee clearance was obtained for carrying out the experiment. They were housed in the animal house of the Pharmacology Department of the college for 7 days for acclimatization in an air conditioned atmosphere at 20°C. Prior to the experiment, all the animals were fasted overnight with water *ad libitum.*

LD_50_ was carried out on mice of either sex according to the Reed and Meuch method[12] and the doses were fixed as 50 mg/kg and 100 mg/kg (i.p.). The BP had no marked effect on the general behavior of rats at the dose of 50 mg/kg (i.p.). However, at the dose level of 100 mg/kg (i.p.), it showed marked depressant effect with the symptoms of auditory and pinna reflex. The depressant effect of BP on CNS was further confirmed by the fact that it had exhibited significant analgesic activity.

### Analgesic activity[13]

**Figure 1:**
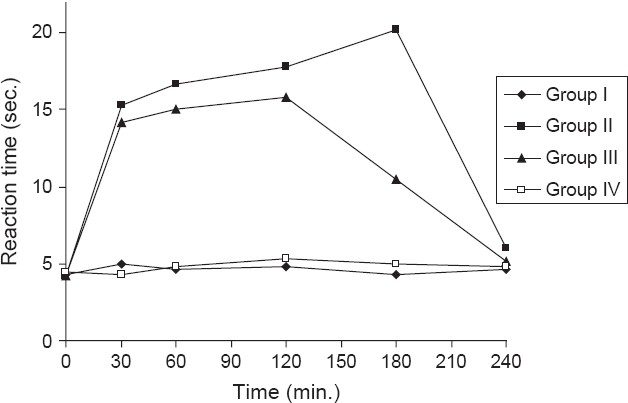
Analgesic activity of ethanol extract of *Bauhinia purpurea* in albino mice

Animal models which showed reaction time of 3-5 seconds were selected for screening of analgesic activity. Albino mice of either sex weighing between 25-30 gm were selected for the experiment and the analgesic activity was studied using Eddy's hot plate method. Mice were divided into four groups of six each and tested for 4 hours. Group I received Tween-80 (1%. i.p.) and served as control. Group II received 150 mg/kg Analgin and served as standard. Group III and group IV received the ethanol extract of the stem of BP at the doses of 50 mg/kg (i.p.) and 100 mg/kg (i.p.) respectively. The observations were made at 30 min intervals up to 4 hours. The results are shown in Table 3 and graphical representation of the results is indicated in Figure 1.

**Table 3:** Analgesic activity of ethanol extract of *Bauhinia purpurea* Linn in albino mice (n = 6)

*Group*	*Reaction time in seconds*					
	
	*0 min*	*30 min*	*60 min*	*120 min*	*180 min*	*240 min*
I	4.33 ± 0.21	5.00 ± 0.0	4.67 ± 0.21	4.83 ± 0.17	4.33 ± 0.21	4.67 ± 0.33
II	4.17 ± 0.17	15.33 ± 0.2	16.67 ± 0.67	17.83 ± 0.65	20.17 ± 0.98	6.00 ± 0.82
III	4.17 ± 0.17	14.17 ± 1.0	15.00 ± 1.06	15.83 ± 0.79	10.50 ± 0.50	5.17 ± 0.17
IV	4.50 ± 0.22	4.33 ± 0.21	4.83 ± 0.17	5.33 ± 0.21	5.00 ± 0.26	4.83 ± 0.17
One way F	0.68	115.6	100.4	165.7	162.1	1.69
ANOVA *P*	0.58 NS	<0.001 HS	<0.001 HS	<0.001 HS	<0.001 HS	0.20 NS

Values (mean ± SE) One-Way ANOVA; Newman-Keul's Test; *P* < 0.01 = S; *P* < 0.001 = HS, *P* > 0.05 = NS

Analgesic activity of the ethanol extract of the stem of BP was also studied by acetic acid induced abdominal constriction (writhing)[14,15] method in mice.

Colony bred Swiss mice of either sex, weighing between 22-25 gm were used to evaluate analgesic activity. Four groups (6 animals each) were injected intraperitoneally with 0.6% acetic acid at the dose of 10 ml/kg and number of writhings was recorded after 5 min for a period of 20 min. All these groups served as control and the same animals were used next day for the evaluation. Animals in group I were given orally Tween-80 (0.5 ml/kg) [control], group II Aspirin (150 mg/kg) [standard], groups III and IV respectively given the extract at the dose of 50 mg/kg and 100 mg/kg. Animals in all the groups were injected with acetic acid (0.6%) intraperitoneally after 1 hour of drug administration; the number of writhings was recorded after 5 min for 20 min and the results are shown in Table 4. Percentage protection was calculated using the formula (1 − Vc/Vt) × 100.

**Table 4:** Analgesic activity of ethanol extract of *Bauhinia purpurea* Linn in albino mice by writhing method (n = 6)

*Group*	*Drug*	*Dose (mg/kg)*	*Mean no. of writhings occurred between 5 and 15 min ± SEM*		*% MPE*
I	Control	5 ml water	45.83 ± 1.28	-	-
II	Aspirin	150	-	08.50 ± 1.1*	81.45
III	Ethanol extract	50	-	22 16 ± 1.9*	51.65
IV	Ethanol extract	100	-	11.83 ± 1.3*	74.19

* *P* < 0.001 vs. control; Student's ‘t’ test

### Anti-inflammatory activity[16]

**Figure 2:**
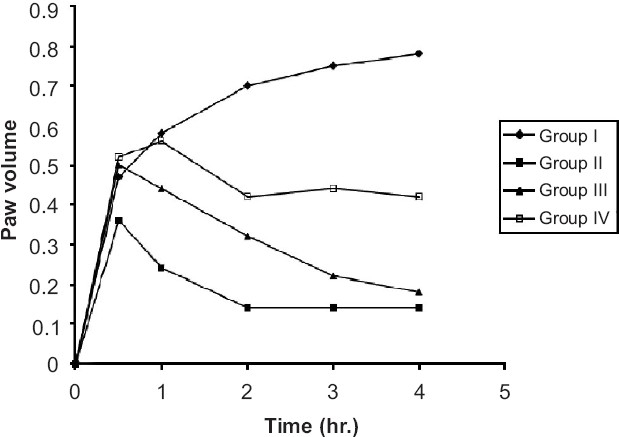
Anti-inflammatory activity of ethanol extract of *Bauhinia purpurea* on carrageenan-induced paw edema in albino rats

**Figure 3:**
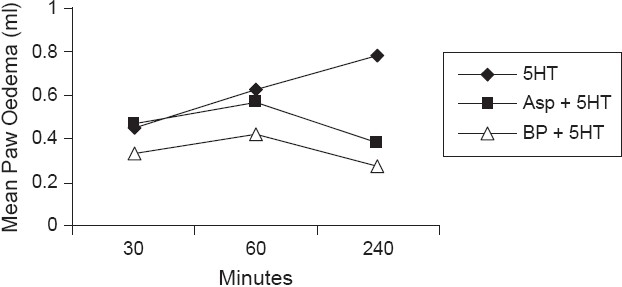
Effect of ethanol extract of *Bauhinia purpurea* on mediator-induced paw edema in albino rats

Twenty-four albino Wistar rats of either sex weighing between 150-200 gm were divided into four groups. Group I received Tween-80 (1%, i.p.) and served as control. Group II received Diclofenac sodium 5 mg/kg and served as standard. Group III and group IV received the ethanol extract of the stem of BP at the doses of 50 mg/kg (i.p.) and 100 mg/kg (i.p.) respectively. One hour after the administration (as per the experimental protocol), 0.1 ml of 1% carrageenan solution was injected beneath the sub-plantar surface of the right hind paw of all animals. For the assessment of the anti-inflammatory activity, the volume of the paw was measured with the help of mercury plethysmometer at 0 h and at 1 h interval for a period of three hours after the carrageenan treatment. The results are tabulated in Tables 5 and 6 and represented graphically in Figures 2 and 3.

**Table 5:** Anti-inflammatory activity of ethanol extract of *Bauhinia purpurea* Linn on carrageenan induced paw edema in albino rats

*Treatment*	*Time, Mean paw volume ± SEM, % edema inhibition*				
	
	*30 min.*	*1 hour*	*2 hours*	*3 hours*	*4 hours*
Control	0.47 ± 0.02	0.58 ± 0.02	0.70 ± 0.04	0.75 ± 0.03	0.78 ± 0.02
Standard	0.36 ± 0.0	0.25 ± 0.02	0.14 ± 0.02	0.14 ± 0.02	0.14 ± 0.02
	-	58.9	80.0	81.3	82.1
Ethanol extract	0.52 ± 0.02	0.56 ± 0.02	0.42 ± 0.02	0.44 ± 0.02	0.42 ± 0.02
(50 mg/kg)	-	4.0	40.0	41.3	46.4
Ethanol extract	0.50 ± 0.03	0.44 ± 0.02	0.32 ± 0.02	0.22 ± 0.02	0.18 ± 0.02
(100 mg/kg)	-	24.6	54.3	70.7	77.0
One-way F	6.03	57.11	89.11	129.80	275.00
ANOVA *P*	0.01 S	0.001 HS	0.001 HS	0.001 HS	0.001 HS

N = 6 Values mean ± SE; One Way ANOVA; Newman-Keul's Test, *P* < 0.01 = S, *P* < 0.001 = HS

**Table 6:** Effect of *Bauhinia purpurea* Linn on mediator –induced edema in rat paw (n = 6)

*Group*	*Treatment*	*Mean changes in paw edema ± SEM*				
		
		*30 min*	*1 hr*	*% Inhibition*	*4 hrs*	*% Inhibition*
I	5HT	0.467 ± .021	0.633 ± 0.021	___	0.783 ± 0.017	__
II	5HT + Aspirin	0.333 ± 0.033	0.417 ± 0.017	34.2	0.267 ± 0.021	65.9
III	5HT + BP	0.450 ± 0.034	0.567 ± 0.056	10.5	0.383 ± 0.017	57.1
One-way	F	5.81	9.63		220.7	
ANOVA	*P*	<0.05 S	<0.05 S		<0.001 HS	

One-Way ANOVA, Newman-Keul's Test; *P* < 0.05 = S; *P* < 0.001 = HS

Albino Wistar rats of either sex weighing between 150-200 gm were divided into three groups (6 animals each). Group I received 5-HT (1 mg/kg, i.p.). Animals in group II and group III received aspirin (20 mg/kg, i.p.) and B.P (100 mg/kg, i.p.) respectively, half an hour prior to the administration of 5-HT solution. The volume of the paw was measured with the help of plethysmometer at 30 min and 1 hr after the 5-HT treatment. The results are reported in Table 6.

## Results and Discussion

### Analgesic activity

Ethanol extract of BP exhibited maximum analgesic activity at 120 min. at 100 mg (i.p.), (*P* < 0.01) and it was significant when compared with control group, but slightly less when compared with standard. In the present study, the ethanol extract of BP was found to possess good analgesic activity at dose of 100 mg/kg (i.p.) as compared to 50 mg/kg (i.p.) Any injury or tissue damage is associated with pain and inflammation. Analgesics can act on peripheral or central nervous system. Peripherally acting analgesics act by blocking the generation of impulses at chemoreceptor site of pain, while centrally acting analgesics not only raise the threshold for pain, but also alter the physiological response to pain and suppress the patient's anxiety and apprehension. Pain and inflammation are an essential prelude to the repair process. The ethanol extract of BP exhibited potent analgesic effect against thermal noxious stimuli. This is evidenced as it exhibited good analgesic activity at 100 mg/kg (i.p.) dose as compared to control and group IV (*P* < 0.001). This showed that the extract acts as a peripheral analgesic. The analgesic activity is attributed to the reported constituents present in the ethanol extract [Table 3].

### Anti-inflammatory activity

Evaluation of anti-inflammatory activity in rats by carrageenan induced rat paw edema indicated that the ethanol extract of BP reduced paw edema by 46.6% and 77% respectively at doses of 50 mg (i.p.) and 100 mg/kg (i.p.) whereas Diclofenac sodium reduced it by 82.1%. Similarly, ethanol extract of BP reduced 5-HT induced edema (included in the experiment) by 46.4% and 65% respectively at the dose of 100 mg (i.p.) and aspirin and the results were statistically significant (*P* < 0.001). At 4^th^ hour, ethanol extract of BP exhibited excellent anti-inflammatory property at the dose of 100 mg (i.p.). Ethanol extract of BP was found to suppress carrageenan induced edema significantly (46.4% and 77% inhibition, *P* < 0.001). Ethanol extract of BP, at 100 mg/kg (i.p.) dose reduced serotonin (5-HT) induced edema significantly (51% inhibition, *P* < 0.001). However, its action is less effective compared with standard drug, aspirin (65.9% inhibition). It is presumed that the anti-inflammatory activity is due to the combined effect of various phytoconstituents reported for ethanol extract upon phytochemical investigation [Table 2]. Prostaglandins (PG_3_) play a significant role in different phases of inflammatory reactions. PG_3_ elicits pain by direct stimulation of sensory nerve endings and also sensitizes sensory nerve endings to other pain provoking stimuli. Since BP has shown significant analgesic and anti-inflammatory activities, the probable mechanism could be by the inhibition of the PG_3_ synthesis. The anti-inflammatory activity may be due to either inhibition of PG biosynthesis or its anti-prostaglandin effect. Hence, there is a need for further investigation on this aspect. The process of inflammation generally consists of three phases. Dilatation and increased permeability of small blood vessels result in edema, swelling, emigration of leucocytes from venules and capillaries, cellular infiltration and a general mopping up reaction, and proliferation of fibroblast and synthesis of new connective tissue to repair the injury. A number of mediators have been identified that initiate the early development (first phase) of certain experimentally induced inflammatory processes. These are considered to be released in a sequential manner. Thus, there is an initial release of histamine and 5-hydroxytryptamine (5-HT) producing an increased vascular permeability followed by release of kinins, further contributing to the increased vascular permeability and finally, the prostaglandins and slow reacting substances (SRS) are released to maintain the increased vascular permeability reproduced by histamine, 5-HT and kinins. The biochemical events accompanying the second phase are not well understood. Many factors are implicated as the regulators of phagocytosis including calcium chemo toxin, leukocyte promoting factor and complement factor.[17] As the exudative phase of inflammation subsides, the initial stages of the reparative or third phase are set in motion. The fibroblast, which is the dominant cell in the wounded zone, first proliferates then synthesizes extra cellular material including new collagen fibers and acidic mucopolysaccharides, which are laid down to form the new connective tissue matrix.

5-HT, histamine, bradykinin and PG (prostaglandin) have been identified as chemical mediators for carrageenan induced hind paw edema. Table 5 shows that ethanolic extract of B.P (100 mg/kg, i.p.) reduced significantly 5-HT induced edema (*P* < 0.001), therefore, it can be interpreted that BP is antagonistic to 5-HT.[18]
